# Procarcinogen Activation and Mutational Signatures Model the Initiation of Carcinogenesis in Human Urothelial Tissues In Vitro

**DOI:** 10.1016/j.eururo.2020.03.049

**Published:** 2020-08

**Authors:** Simon C. Baker, Andrew S. Mason, Jennifer Southgate

**Affiliations:** a Jack Birch Unit for Molecular Carcinogenesis, Department of Biology, Heslington, York, UK; b York Biomedical Research Institute, University of York, Heslington, York, UK

**Keywords:** APOBEC, BaP, Benzo[a]pyrene, Bladder, Bladder cancer, CYP1A1, Cytochrome P450, DNA mutation, Urothelium, Whole-genome DNA sequencing

## Abstract

Disparity between genome-wide mutations in bladder and other cancers where smoking is a risk factor raises questions about carcinogenesis in different epithelia. To develop an experimental model of bladder carcinogenesis, we clonally expanded in vitro differentiated normal human urothelial (NHU) cells following exposure to an exemplar procarcinogen and used whole-genome DNA sequencing to derive mutational signatures. Benzo[a]pyrene (BaP) was activated by endogenous cytochrome P450 (cytochrome P450 family 1 subfamily A member 1 [CYP1A1]) to create genomically modified NHU cells. Comparison with the Catalogue of Somatic Mutations in Cancer (COSMIC) showed that mutations induced by BaP in NHU cells were similar to smoking-associated signatures in bladder and other cancers, including single- and doublet-base substitution signatures characterised by C > A transversions (COSMIC_SBS4 and COSMIC_DBS2, respectively), and an insertion/deletion signature of C deletions in homopolymer regions (COSMIC ID3). Our study provides the first direct evidence that BaP is activated locally in the urothelium, initiating the well-described smoking-associated mutational signatures. An absence of other common bladder cancer (BLCA)-associated genomic signatures points strongly to other primary causes of BLCA, which the new experimental approach described here is well placed to investigate. Mutational signatures ignore whether genes are affected, but tissue-specific drivers (*KMT2D*, *KMT2C*, and *CDKN1A)* were significantly overmutated in this model, providing insight on the emergent selection pressures.

**Patient summary:**

In a carefully controlled laboratory setting, we exposed normal human urothelial tissues to a procarcinogen (benzo[a]pyrene) found in cigarette smoke. We show that the urothelial tissues activated the carcinogen and led to mutations forming across the genome in a characteristic pattern. This particular “mutational signature” is found in bladder tumours and other smoking-induced cancers (eg, lung); however, our study highlights that there are other unknown mutational processes in bladder cancer that is not the direct result of smoke carcinogens, and this will require further investigation.

## Take Home Message

Clonal expansion and whole-genome sequencing of finite normal human urothelial cells, which retained their native differentiated and quiescent state during exposure to putative bladder carcinogens, provide new avenues for tissue-relevant research into the causes of bladder cancer.

Lifetime accrual of mutations in the cells of our different tissues reflects the interactions between carcinogen exposure(s), genomic damage, tissue-specific gene expression, and the DNA-repair machinery. Whole-genome DNA sequencing (wgDNAseq) identifies the thousands of passenger and rarer driver mutations, with the frequency of each base-change–type relative to its genomic context described as a “mutational signature” [1]. As an aggregated historical record of carcinogen-tissue interactions, tumour mutational signatures are complex and challenging to deconvolute. The potential to combine advances in genomic sequencing with in vitro tissue-specific models offers a timely opportunity to relate controlled carcinogen exposures directly to molecular initiating events for specific cancer types.

**Fig. 1:**
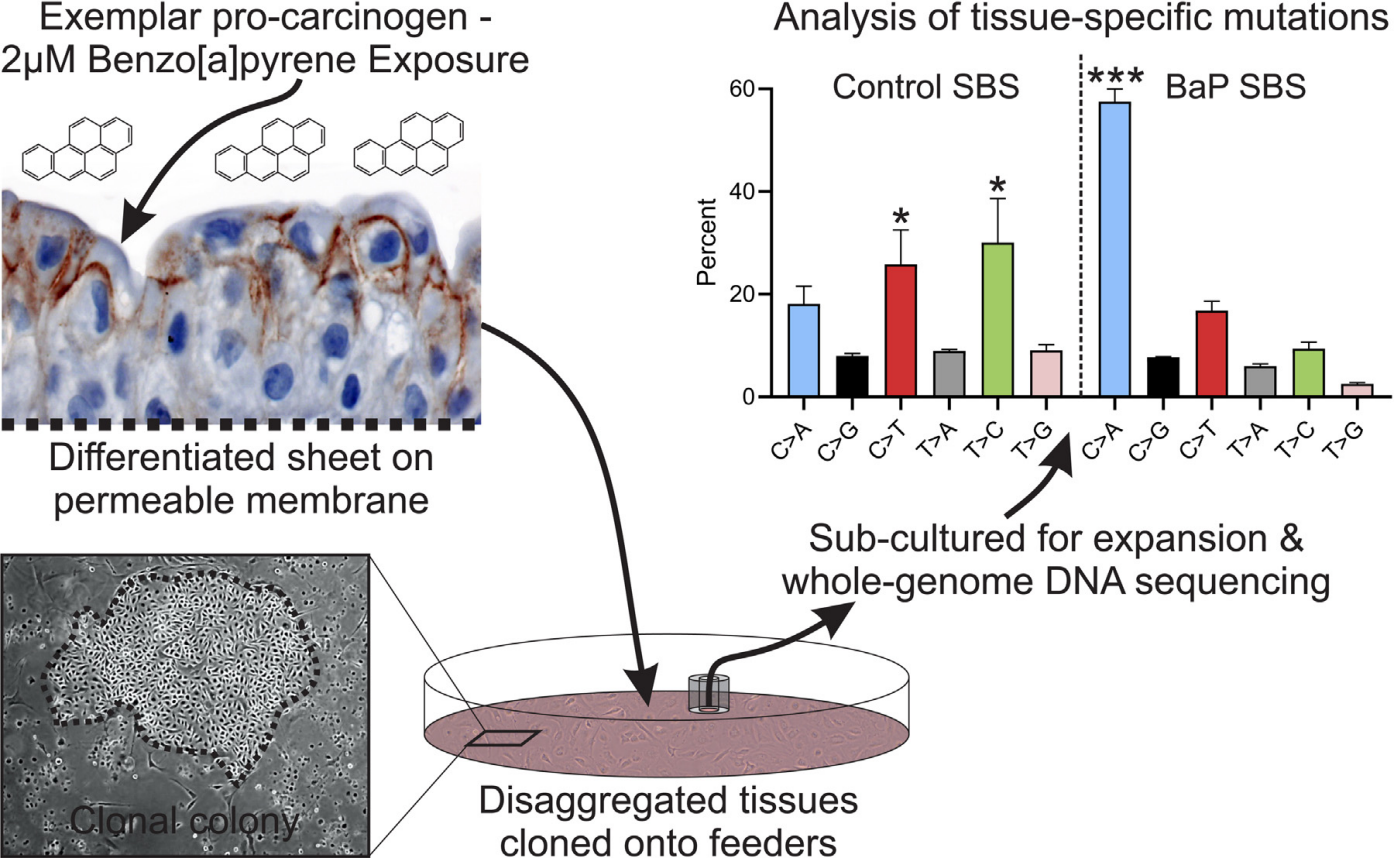
Schematic of the experimental approach wherein functionally differentiated NHU cell sheets (generating a transepithelial electrical resistance of >2000 Ω.cm^2^; Supplementary Fig. 1) were exposed to benzo[a]pyrene (BaP) for 7 d before single-cell clones were expanded as colonies in feeder-supported cultures. Expanded clones were analysed by wgDNAseq and mutational signatures were derived. Graph shows mean percentage of mutations within each class of single-base substitution (SBS). Error bars = standard deviation (*n* = 3, control, and *n* = 4, BaP-exposed independent clones). The control SBS percentages (ANOVA *p* = 0.0002) showed significant enrichment of C > T and T > C transitions, which are more common than transversions in virtually all DNA sequences. In the control SBS, C > A transversions were not significantly enriched. The BaP SBS percentages showed significant enrichment of C > A transversions. Multiple comparisons were performed using Tukey’s post hoc test. ANOVA = analysis of variance; NHU = normal human urothelial; wgDNAseq = whole-genome DNA sequencing. * C > T and T > C changes were significantly enriched (*p* < 0.01) in comparison with C > G, T > A, and T > G. *** *p* < 0.0001 for C > A changes in comparison with all other types.

As a tissue evolved to endure exposure to urinary toxins/toxicants, including carcinogens, urothelium differentiates to form a tight urinary barrier epithelium with an inducible capacity to metabolise xenobiotics [2]. Paradoxically, phase I metabolism may result in the activation of procarcinogens (including 2‐naphthylamine, benzidine, 4,4′-methylenebis(2-chloroaniline), 4‐aminobiphenyl, and benzo[a]pyrene), leading to local initiation of urothelial carcinogenesis, but no experimental system has yet been established to model this scenario. We generated functional barrier-forming differentiated urothelium from isolated, in vitro–propagated normal human urothelial (NHU) cells. Following exposure to 2 μM benzo[a]pyrene (BaP) for 7 d, urothelial cultures remained viable, as reported previously [2] and demonstrated by retention of barrier tightness (Supplementary Fig. 1). After maintenance for a further 7 d, control and exposed tissues were disaggregated and expanded as clones on irradiated 3T3-J2 feeder cells in proliferative cell culture conditions, before genomic analysis to determine mutational signatures (Fig. 1).

**Fig. 2:**
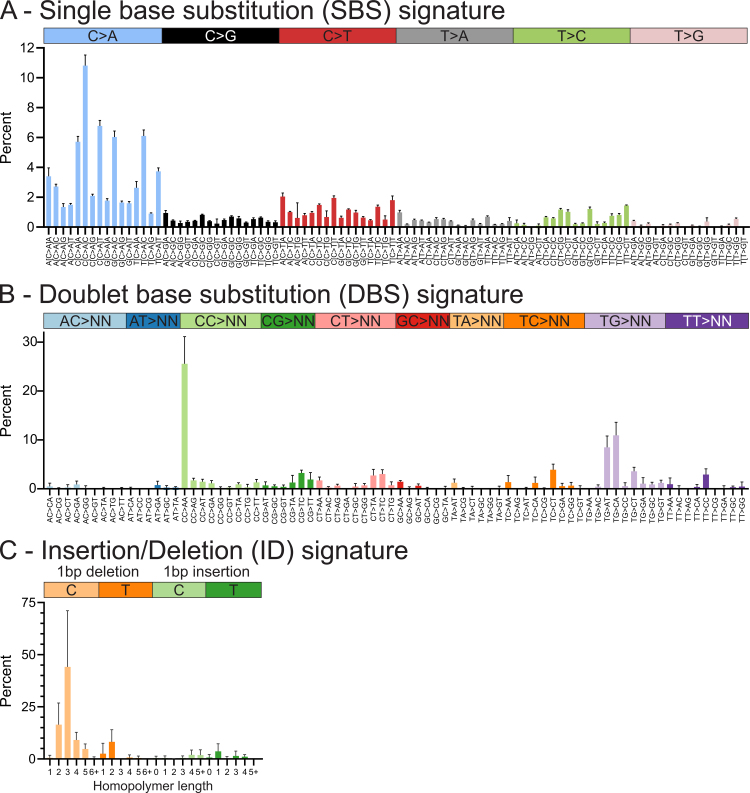
Mutational signatures derived from BaP-exposed functionally differentiated NHU cells. (A) Single-base substitution (SBS) signature of 96 subtypes based around six substitution classes (referred to by the pyrimidine of the mutated Watson-Crick base pair) and framed by their 3′ and 5′ flanking nucleotides. The SBS signature shows an enrichment of diverse C > A transversions. (B) Doublet-base substitution (DBS) signature of 78 strand-agnostic mutation types show an enrichment mainly of CC > AA. TG > AT and TG > CA substitutions were additionally observed and were previously reported following BPDE exposure of iPS cells [5]. (C) Insertions/deletion (ID) signature reveals BaP-caused single C/G deletions most commonly in homopolymer runs of two to four cytosines/guanines (n = 4 independent BaP-exposed clones normalised to n = 3 independent control clones; bars indicate the mean and error bars denote the standard deviation). Data in this figure are expressed as percentages; for counts and normalisation data, see Supplementary Figures 2–4. BaP = benzo[a]pyrene; BPDE = benzo(a)pyrene diol epoxide; iPS = induced pluripotent stem; NHU = normal human urothelial.

NHU cultures thus exposed to BaP showed significantly increased C > A transversions (*p* < 0.0001; Fig. 1); these mutations arise following cytochrome P450 family 1 subfamily A member 1 (CYP1A1)-mediated activation of BaP and formation of guanine DNA adducts [2]. Single nucleotide polymorphisms in *CYP1A1* have been associated with bladder cancer (BLCA) risk [3]. Clear signatures of single-base substitution (SBS) C > A transversions (“SBS-BaP-NHU”; Fig. 2A; derivation detailed in Supplementary Fig. 2) and doublet-base substitution (DBS) CC > AA transversions (“DBS-BaP-NHU”; Fig. 2B and Supplementary Fig. 3) were apparent. In addition, a BaP-induced insertion/deletion (ID) signature of C deletions was found in association with homopolymer regions (“ID-BaP-NHU”; Fig. 2C and Supplementary Fig. 4). No karyotypic abnormalities were observed in BaP-exposed clones.

C > A transversions constitute 10% of mutations in the exome-sequenced BLCA cohort of The Cancer Genome Atlas (TCGA; Supplementary Fig. 5). Analysis of SBS-BaP-NHU using the “signal” workflow [4] and cosine similarity testing allowed comparison with pan-cancer and tissue-specific tumour signatures. Catalogue of Somatic Mutations in Cancer (COSMIC)_SBS4, the “smoking signature” found in tissues exposed directly to tobacco smoke [1] and ascribed experimentally to BaP [5], was found to be the closest match in the largest pan-cancer database [1] (Supplementary Fig. 6 and 7). COSMIC_SBS4 has been observed in a wgDNAseq BLCA cohort (*n* = 85 [6]), although not that of the COSMIC study itself (*n* = 23 [1]). COSMIC signatures SBS4, DBS2, and ID3 are frequently found together as a triad of smoke-induced mutations [1]. DBS-BaP-NHU and ID-BaP-NHU, which correspond directly to COSMIC signatures DBS2 and ID3, are found in most BLCA (Supplementary Fig. 8 [1]). C > A dominated signatures have been detected de novo in BLCA, of which “Bladder_F” was most similar to SBS-BaP-NHU and is closely related to the smoking signatures of other tissues [4], [7] (Supplementary Fig. 6 and 7).

Our study supports a role for BaP in bladder carcinogenesis. BaP is a polycyclic aromatic hydrocarbon (PAH) procarcinogen found in cigarette smoke and detectable in the urine of smokers or healthy volunteers after a PAH-rich meal [8]. Cigarette smoking is the main risk factor for BLCA (estimated hazard ratio is 2–4 for current smokers [9]).

Our results support the capacity of urothelium to activate BaP locally, initiating BLCA-relevant mutations within the epithelium. Such initiated cells were retained viable within the barrier urothelium and capable of clonal expansion in a proliferative setting. This attests to the potential for carcinogen-initiated cells to remain nascent within the tumour-suppressive environment of the mitotically quiescent urothelium until promoted by a regenerative signal. A recent study of induced pluripotent stem (iPS) cells exposed to either 2 µM BaP (with metabolic activation provided by rat liver S9 fraction) or the adduct-forming metabolite benzo(a)pyrene diol epoxide (BPDE; 0.125 µM) [5] found similar signatures to SBS-BaP-NHU with both exposures (Supplementary Fig. 7). However, the changes observed in DBS-BaP-NHU and ID-BaP-NHU were observed only in iPS cells exposed to BPDE, and not BaP + S9 [5]. These results combine with the detection of the similar COSMIC signatures DBS2 and ID3 in BLCA [1], [6], to support a fundamental role for local procarcinogen activation by urothelium in bladder carcinogenesis.

Mutational signatures specifically ignore the location of observed mutations, but tissue-specific carcinogenesis is critically reliant on where specific driver mutations occur. To establish the disease relevance of this model, we interrogated the location of mutations observed in relation to areas of active transcription. Linear regression indicated a significant increase in mutation rate associated with areas of active transcription (*p* < 0.0001; Supplementary Fig. 9). Tumour studies have described an inverse correlation between gene expression and mutation rate [10]. Hypothetically, this is due to efficient transcription-coupled DNA repair (“TCR”), although it is possible that the evolutionary selection of tumour cells favours those with enhanced TCR. We selected genes frequently mutated in TCGA BLCA cohort to perform a focussed analysis in BaP-exposed NHU cells (Supplementary Table 1). Whilst most genes gained mutations in a pattern consistent with a random distribution of mutations across the genome, *KMT2D*, *KMT2C*, and *CDKN1A* were significantly overmutated, whilst *PIK3CA*, *RB1*, *ATM*, *KMT2A*, *ASXL2*, and *FBXW7* were significantly protected from mutation (Supplementary Table 1). This provides novel evidence of the gene (and hence driver) selection pressures operating on initiated (BaP-exposed) cells during proliferative expansion (promotion).

Whilst our BaP-exposure study found the canonical smoking signatures, it did not find evidence for COSMIC signatures SBS2/SBS13, which account for most mutations in BLCA and are ascribed to apolipoprotein B mRNA editing enzyme catalytic polypeptide-like (APOBEC) activity [1], [6]. This lack of evidence of APOBEC activity strongly suggests that carcinogen-induced genomic stress per se (and BaP specifically) does not lead to persistence of single-stranded genomic DNA sufficient to trigger APOBEC-mediated mutagenesis in the tissue and predicts an additional mechanism. The new experimental system described here is well placed to address the question of APOBEC activation and other gaps in our understanding of BLCA aetiopathology.

  ***Author contributions:*** Simon C. Baker had full access to all the data in the study and takes responsibility for the integrity of the data and the accuracy of the data analysis.

*Study concept and design:* Baker.

*Acquisition of data:* Baker.

*Analysis and interpretation of data:* Mason, Baker.

*Drafting of the manuscript:* Baker, Mason.

*Critical revision of the manuscript for important intellectual content:* Baker, Mason, Southgate.

*Statistical analysis:* Mason, Baker.

*Obtaining funding:* Baker, Southgate.

*Administrative, technical, or material support:* None.

*Supervision:* None.

*Other:* None.

  ***Financial disclosures:*** Simon C. Baker certifies that all conflicts of interest, including specific financial interests and relationships and affiliations relevant to the subject matter or materials discussed in the manuscript (eg, employment/affiliation, grants or funding, consultancies, honoraria, stock ownership or options, expert testimony, royalties, or patents filed, received, or pending), are the following: None.

  ***Funding/Support and role of the sponsor:*** This study was funded by York Against Cancer and Department of Biology Pump-Priming funding. Simon C. Baker was also supported in part by the 10.13039/100004440Wellcome Trust (092430/Z/10/Z) and 10.13039/100009150Centre for Chronic Diseases and Disorders (C2D2; award 105624).

  ***Acknowledgements:*** The authors would like to thank Dr. Serena Nik-Zainal (University of Cambridge) for her support throughout the project and Dr John Davey (Bioscience Technology Facility, University of York) for bioinformatics assistance. Data on wgDNAseq have been deposited at https://www.ncbi.nlm.nih.gov/sra (SRA accession: SAMN14260674, SAMN14260675, SAMN14260676, SAMN14260677, SAMN14260678, SAMN14260679 and SAMN14260680; embargoed until publication). Data on mRNAseq have been deposited at https://www.ncbi.nlm.nih.gov/geo/ (GEO accession: GSE146372; embargoed until publication).

## CRediT authorship contribution statement

**Simon C. Baker:** Conceptualization, Methodology, Software, Data curation, Writing - original draft, Visualization, Project administration, Funding acquisition. **Andrew S. Mason:** Software, Data curation, Writing - review & editing. **Jennifer Southgate:** Writing - review & editing, Funding acquisition.
