# Supplementary material for: Procarcinogen Activation and Mutational Signatures Model the Initiation of Carcinogenesis in Human Urothelial Tissues In Vitro
Source: Eur Urol. 2020 Aug;78(2):143–7. doi: 10.1016/j.eururo.2020.03.049 (PMC7397502; doi:10.1016/j.eururo.2020.03.049)
Supplement: Supplementary file 1 [file mmc1.docx]

**Supplementary material**

**Methods**

A graphical summary of the method is provided as Fig. 1. Unless otherwise stated laboratory reagents were sourced from Merck (Gillingham, UK).

***The Cancer Genome Atlas exome DNA sequencing mutation data***

The Cancer Genome Atlas (TCGA) mutation data were downloaded from the National Cancer Institute Genomic Data Commons dbGaP accession phs000178.v10.p8 [1] as part of dbGaP project 19625.

***Normal human urothelial cell culture***

Normal human urothelial (NHU) cells were established and maintained, as previously described [2], in keratinocyte serum-free medium (KSFM, Thermo Fisher Scientific, Loughborough, UK) supplemented with bovine pituitary extract, recombinant human epidermal growth factor, and cholera toxin (referred to as “complete medium” or KSFMc). Following expansion in KSFMc, NHU cells were differentiated by serum exactly according to existing methods [3].

***Transepithelial electrical resistance monitoring***

The transepithelial electrical resistance (TEER) of NHU cell cultures grown on permeable polyethylene terephthalate (PET) membranes (ThinCert cell culture inserts; Greiner, Stonehouse, UK) was monitored as a measure of differentiated barrier function using STX2 chopstick electrodes and epithelial volt/ohm meter (EVOM; World Precision Instruments, Hitchin, UK). The probe was sterilised using Rapicide (World Precision Instruments); blank membranes were used to establish baseline resistances, and all measurements were corrected for membrane area.

***Benzo[a]pyrene exposure***

Benzo[a]pyrene (BaP; IARC classification group 1 “carcinogenic to humans”; B1760) exposure was performed exactly as previously described for inducing adducts at a concentration of 2 µM, which induces no acute cytotoxicity in differentiated NHU cells [4]. Differentiated NHU cells cultured on PET membranes were exposed to either 2 µM BaP or vehicle control (0.1% dimethyl sulfoxide) for 7 d, with medium changed every 2–3 d. Following 7 d of exposure, cells were returned to KSFMc with 5% serum and adjusted to 2 mM [Ca^2+^] for 6 d.

***Feeder cell culture***

Swiss 3T3 J2 feeder cells (Kerafast, Boston, MA, USA) were expanded in Dulbecco’s Modified Eagle Medium (Thermo Fisher Scientific, Loughborough, UK) supplemented with 10% (v/v) bovine calf serum (BCS) and 100 µM sodium ascorbate. To support the cloning of NHU cells, ~90% confluent feeder cell cultures were irradiated (30 Gy) on the morning of use and washed once in Dulbecco's phosphate-buffered saline (D-PBS; Thermo Fisher Scientific, Loughborough, UK) prior to use. An RS2000 x-ray biological irradiator was used, which contains a Comet MXR-165 x-ray source (Rad-Source Technologies Inc., Buford, GA, USA).

***NHU cell cloning***

After BaP exposure and washout, cell cultures on PET membranes were washed once in D-PBS and cultured in cloning medium for 24 h. Cloning medium (KSFMc, 10 µM Y27632, 3 µM SB431542, 0.25% [v/v] BCS) was based on previous studies [5], with adaptations to improve cloning efficiency.

To clone differentiated NHU cells, sheets were first released intact from the PET membranes by incubation in 0.5% (w/v) dispase II solution in PBS for 1 h at 37°C. Urothelial sheets were disaggregated into single-cell suspensions by serial incubation in EDTA (5 min) and trypsin/versene (1 min) at 37°C. Trypsin was neutralised using soybean trypsin inhibitor, and cells were counted for seeding in 10 cm Cell+ dishes (Sarstedt) at colony-forming density (~300 cells/cm^2^) on washed, irradiated 3T3 J2 cells in cloning medium.

Mixed NHU/feeder cells were cultured for 7 d without medium change to establish colonies. After 7 d, medium was changed to cloning medium without SB431542 to enhance proliferation, and after 5 d, visible colonies were ready for passage (shown in Supplementary Fig. 10).

The end of a borosilicate glass cloning cylinder (internal diameter 3 mm × height 8 mm) was coated in sterile vacuum grease and used to isolate selected colonies. Colonies were passaged in EDTA (5 min) and trypsin/versene (5 min) at 37°C. Cells were pipetted vigorously in trypsin/versene to generate a single-cell suspension prior to trypsin neutralisation by soybean inhibitor. Passaged colonies were seeded into individual wells of a 24-well plate on washed 3T3 J2 cells in KSFMc with 10 µM Y27632 and 0.25% (v/v) BCS. Medium of clonal cultures were changed on alternate days and harvested by passaging at confluence. Passaged clones were pelleted and frozen dry at –80°C for subsequent DNA extraction.

***Whole genome DNA sequencing***

DNA was extracted from cell pellets using Macherey-Nagel NucleoSpin TissueXS kit (Fisher Scientific, Loughborough, UK) according to instructions, and contaminating RNA was removed by RNase A digestion (5 mg/ml for 5 min at ambient temperature). Selected clones (three control and four BaP exposed) with sufficient DNA were sent to Novogene Corporation Limited (Cambridge, UK) for library preparation and whole-genome resequencing. Sequencing libraries were generated using NEBNext DNA Library Prep Kit according to the manufacturer's recommendations (New England Biolabs, Hitchin, UK). Genomic DNA was randomly fragmented to 350 base pairs (bp) by shearing. DNA fragments were end polished, A tailed, and ligated with the NEBNext adapter, and further enriched with polymerase chain reaction (PCR) by P5 and indexed P7 oligos. PCR products were purified (AMPure XP system; Beckman Coulter, High Wycombe, UK). Libraries were verified by 2100 Bioanalyzer (Agilent, Cheadle, UK) and quantified using real-time PCR. Whole genome resequencing data (150 bp, paired end reads) were generated using NovaSeq6000 (Illumina, Cambridge, UK; all samples gained >300 million reads with Q30 >91%). Raw data can be found at the National Center for Biotechnology Information (NCBI) Sequence Read Archive (SRA) https://www.ncbi.nlm.nih.gov/sra under the accession numbers SAMN14260674, SAMN14260675, SAMN14260676, SAMN14260677, SAMN14260678, SAMN14260679, and SAMN14260680.

***Variant calling***

Data were checked for quality using FastQC [6] v0.11.7, and no trimming or adapter removal was required. Reads were aligned to the human reference genome (GenBank: GCF_000001405.38) with Minimap2 [7] v2.12 using the short read preset. Alignment maps were processed following GATK best practices [8] using GATK [9] v4.1.0.0, PicardTools [10] v2.20.2, and SAMtools [11] v1.9. Candidate single-nucleotide variants (SNVs) were called with BCFtools [12] v1.9 using the multiallelic caller, skipping indels and requiring base quality ≥30 and read mapping quality ≥20. Called SNVs shared between at least two control samples were removed from all samples. SNVs with quality ≥15 and read depth of 10–50 inclusive were retained for signature analysis. Indels were called using Pindel [13] v0.2.5b9 with default settings.

Genes commonly mutated in TCGA cohort of bladder cancer [14] were inspected for their mutational burden following BaP exposure. To assess whether observed burden fell outside that of a model of random mutation, an equal number of mutations were randomly modelled across the genome using BEDTools [15] v2.27.1, repeated 100 000 times. Observed burden was compared with the model using a binomial test.

***Mutational signature derivations***

All SNVs were considered as either a reference C or a reference T allele in a strand-independent manner, considered in respect to the 5′ and 3′ bases to form 96 single-base substitution (SBS) triplets and 78 types of doublet-base substitution (DBS). DBS mutations were excluded from the SBS signature, and triple-base and higher SNVs were excluded from all analyses. In a similar manner, the insertion/deletion (ID) signature was generated following the Catalogue of Somatic Mutations in Cancer (COSMIC) single-base indel signatures, considering insertion or deletion of C or T within homopolymers of that base. Counts were taken for each substitution type, and the maximum count value observed in control samples was subtracted from each of the four BaP-exposed samples to generate a BaP signature (negative values were corrected to zero).

Graphs showing count data and the effect of normalisation on the BaP SBS, DBS, and ID signatures are included as Supplementary Figures 2–4, respectively. Numerical percentage data for the SBS, DBS, and ID signatures for control and BaP-exposed clones are included as Supplementary Tables 2–4.

The SBS signature was analysed using the “Catalog” input for the “signal” workflow for mutational signature analysis (https://signal.mutationalsignatures.com/ [16]).

Larger structural variants were examined using Genomic Rearrangement IDentification Software Suite (GRIDSS) [17] v2.7.2. No major karyotypic events were detected in the BaP-exposed clones (data not shown).

***Cosine similarity testing***

Mutational signatures were converted from counts to percentages to allow direct comparison with signatures observed in pan-cancer studies (COSMIC signatures [18] “May 2019 v3” were downloaded from Synapse SBS = syn11738319, DBS = syn11738317, ID = syn11738318), bladder cancer–specific signatures [16,19], and induced pluripotent stem (iPS) cells exposed to BaP/benzo(a)pyrene diol epoxide (BPDE) [20]. Cosine similarity tests were performed in scikit-learn [21] v0.20.3 (implemented in Python v3.7). A value of 1 × 10^–10^ was added to any zero values for a substitution type within a signature to prevent aberrant test results, and mean cosine similarity across the signature is reported for comparison.

***Association of mutations with transcriptional activity***

Finite (nonimmortalised) NHU cell lines from three independent donors were differentiated on membranes according to the method described above, reaching the mean TEER of 3757 Ω.cm^2^ (±206 standard deviation). RNA was harvested in TRIzol. Sequencing of polyadenylated mRNA was performed by Illumina HiSeq2000 (100 bp, paired-end reads; with data deposited at https://www.ncbi.nlm.nih.gov/geo/, accession number GSE146372). The quality of reads was checked using FastQC. Retained adapter sequences were removed and reads were trimmed using Trimmomatic [22] v0.36 when the 4-bp sliding-window average base quality dropped below 20, and reads dropped if over half of the original reads were trimmed. Remaining reads were “pseudoaligned” to the Ensembl GRCh38.p10 reference transcriptome using kallisto [23] v0.46.0, and the expression at gene level was expressed using tximport [24] v1.14.0 in R v3.6.0. Data were expressed as transcripts per million (TPM), and the mean TPM was analysed against the BaP-induced mutation rate for every gene by linear regression.

**References**

[1] Grossman RL, Heath AP, Ferretti V, et al. Toward a shared vision for cancer genomic data. N Engl J Med 2016;375:1109–12.

[2] Southgate J, Hutton KA, Thomas DF, Trejdosiewicz LK. Normal human urothelial cells in vitro: proliferation and induction of stratification. Lab Invest 1994;71:583–94.

[3] Cross WR, Eardley I, Leese HJ, Southgate J. A biomimetic tissue from cultured normal human urothelial cells: analysis of physiological function. Am J Physiol Renal Physiol 2005;289:F459–68.

[4] Baker SC, Arlt VM, Indra R, et al. Differentiation-associated urothelial cytochrome P450 oxidoreductase predicates the xenobiotic-metabolizing activity of "luminal" muscle-invasive bladder cancers. Mol Carcinog 2018;57:606–18.

[5] Liu X, Ory V, Chapman S, et al. ROCK inhibitor and feeder cells induce the conditional reprogramming of epithelial cells. Am J Pathol 2012;180:599–607.

[6] Andrews S. FastQC: a quality control tool for high throughput sequence data. 2010. https://wwwbioinformaticsbabrahamacuk/projects/fastqc/

[7] Li H. Minimap2: pairwise alignment for nucleotide sequences. Bioinformatics 2018;34:3094–100.

[8] DePristo MA, Banks E, Poplin R, et al. A framework for variation discovery and genotyping using next-generation DNA sequencing data. Nat Genet 2011;43:491–8.

[9] McKenna A, Hanna M, Banks E, et al. The Genome Analysis Toolkit: a MapReduce framework for analyzing next-generation DNA sequencing data. Genome Res 2010;20:1297–303.

[10] Broad I. Picard Toolkit. 2019. http://broadinstitutegithubio/picard/

[11] Li H, Handsaker B, Wysoker A, et al. The Sequence Alignment/Map format and SAMtools. Bioinformatics 2009;25:2078–9.

[12] Li H. A statistical framework for SNP calling, mutation discovery, association mapping and population genetical parameter estimation from sequencing data. Bioinformatics 2011;27:2987–93.

[13] Ye K, Schulz MH, Long Q, Apweiler R, Ning Z. Pindel: a pattern growth approach to detect break points of large deletions and medium sized insertions from paired-end short reads. Bioinformatics 2009;25:2865–71.

[14] Robertson AG, Kim J, Al-Ahmadie H, et al. Comprehensive molecular characterization of muscle-invasive bladder cancer. Cell 2017;171:540–56 e25.

[15] Quinlan AR, Hall IM. BEDTools: a flexible suite of utilities for comparing genomic features. Bioinformatics 2010;26:841–2.

[16] Degasperi A, Amarante TD, Czarnecki J, et al. A practical framework and online tool for mutational signature analyses show intertissue variation and driver dependencies. Nat Cancer 2020;1:249–63.

[17] Cameron DL, Schroder J, Penington JS, et al. GRIDSS: sensitive and specific genomic rearrangement detection using positional de Bruijn graph assembly. Genome Res 2017;27:2050–60.

[18] Alexandrov LB, Kim J, Haradhvala NJ, et al. The repertoire of mutational signatures in human cancer. Nature 2020;578:94–101.

[19] Fantini D, Seiler R, Meeks JJ. Molecular footprints of muscle-invasive bladder cancer in smoking and nonsmoking patients. Urol Oncol 2019;37:818–25.

[20] Kucab JE, Zou X, Morganella S, et al. A compendium of mutational signatures of environmental agents. Cell 2019;177:821–36 e16.

[21] Pedregosa F, Varoquaux G, Gramfort A, et al. Scikit-learn: machine learning in Python. J Mach Learn Res 2011;12:2825–30.

[22] Bolger AM, Lohse M, Usadel B. Trimmomatic: a flexible trimmer for Illumina sequence data. Bioinformatics 2014;30:2114–20.

[23] Bray NL, Pimentel H, Melsted P, Pachter L. Near-optimal probabilistic RNA-seq quantification. Nat Biotechnol 2016;34:525–7.

[24] Soneson C, Love MI, Robinson MD. Differential analyses for RNA-seq: transcript-level estimates improve gene-level inferences. F1000Res 2015;4:1521.

[25] Fromter E, Diamond J. Route of passive ion permeation in epithelia. Nat New Biol 1972;235:9–13.

[26] Yang Z, Yoder AD. Estimation of the transition/transversion rate bias and species sampling. J Mol Evol 1999;48:274–83.

[27] Priestley P, Baber J, Lolkema MP, et al. Pan-cancer whole-genome analyses of metastatic solid tumours. Nature 2019;575:210–6.


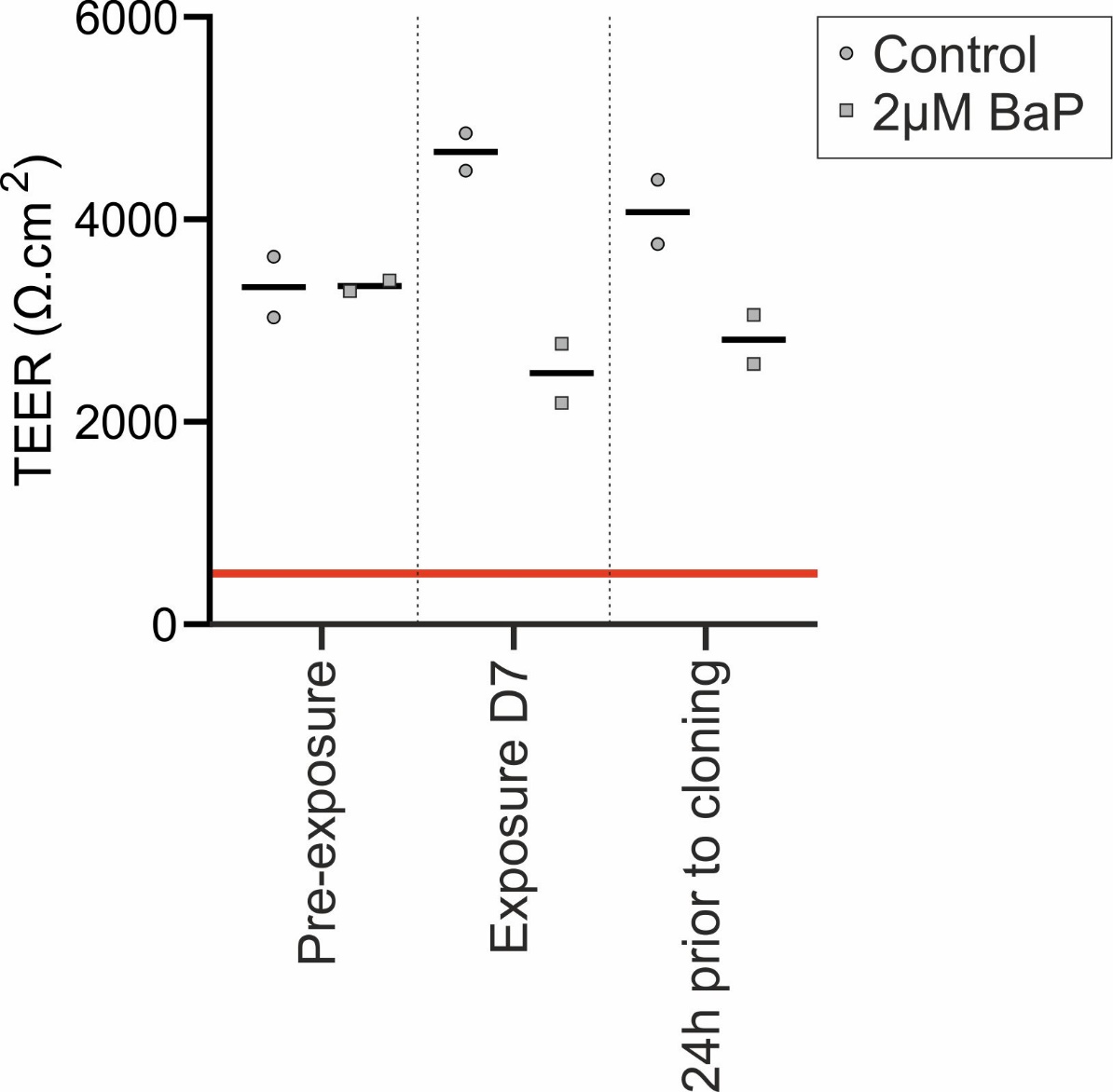


**Supplementary Fig. 1 – TEER data from NHU cells before treatment after 7 d of BaP exposure and 24 h prior to cloning. The red line indicates the literature threshold for a tight barrier (500 Ω.cm^2^ [25]). BaP = benzo[a]pyrene; NHU = normal human urothelial; TEER = transepithelial electrical resistance.**

*
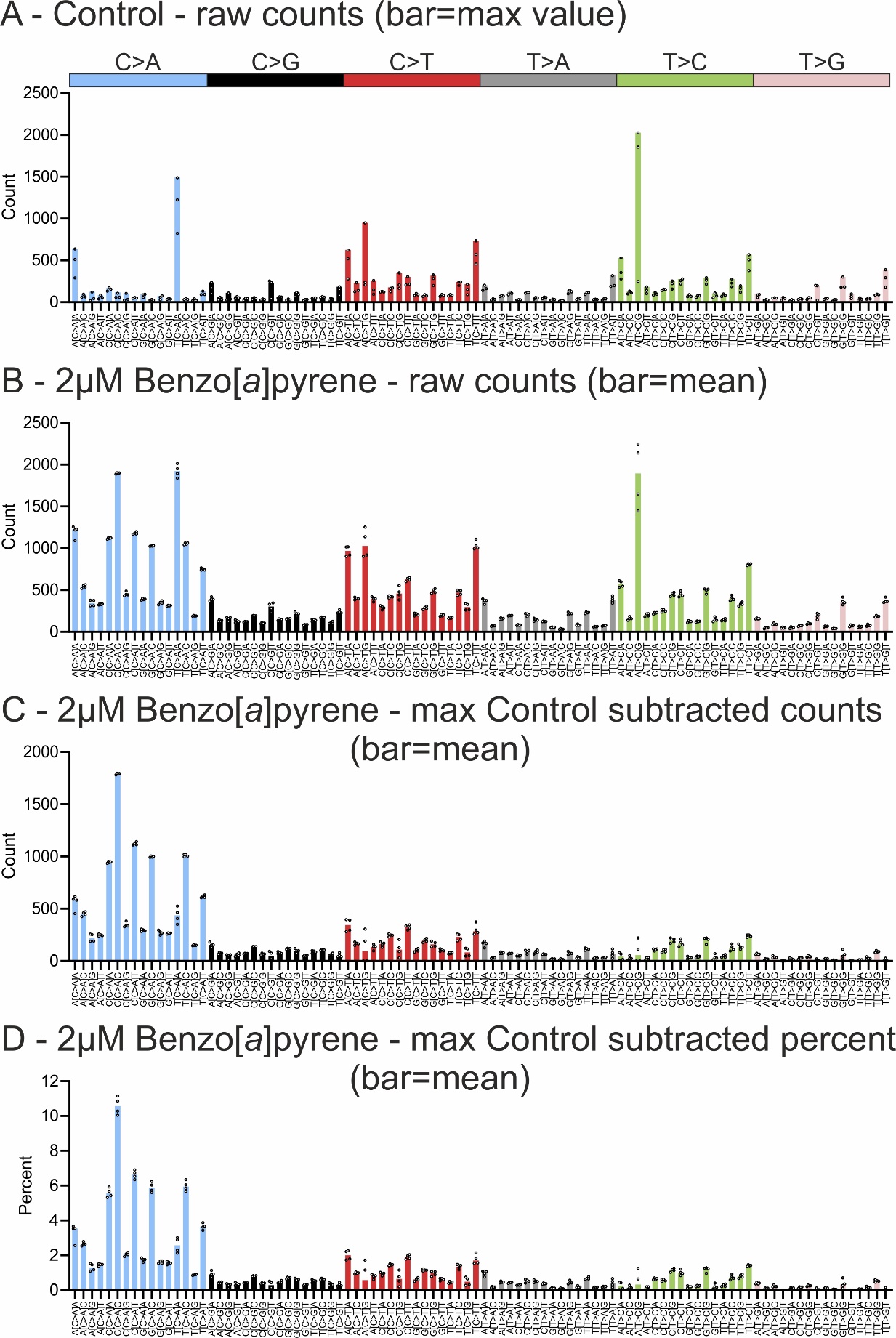
*

**Supplementary Fig. 2 – Counts and normalisation data for the SBS-BaP-NHU signature. Dots indicate the experimental replicates. The raw counts of the different mutation types are shown for (A) control and (B) BaP-exposed clones. (C) The counts from Supplementary Figure 2B with the maximum count from Supplementary Figure 2A subtracted. (D) Supplementary Figure 2C converted to percentage for comparison with other studies including COSMIC_SBS# [18]. The control signature shows a higher rate of C > T and T > C transitions, which are more common than transversions in nearly all DNA sequences due their chemistry [26]. BaP = benzo[a]pyrene; COSMIC = Catalogue of Somatic Mutations in Cancer; NHU = normal human urothelial; SBS = single-base substitution.**

*
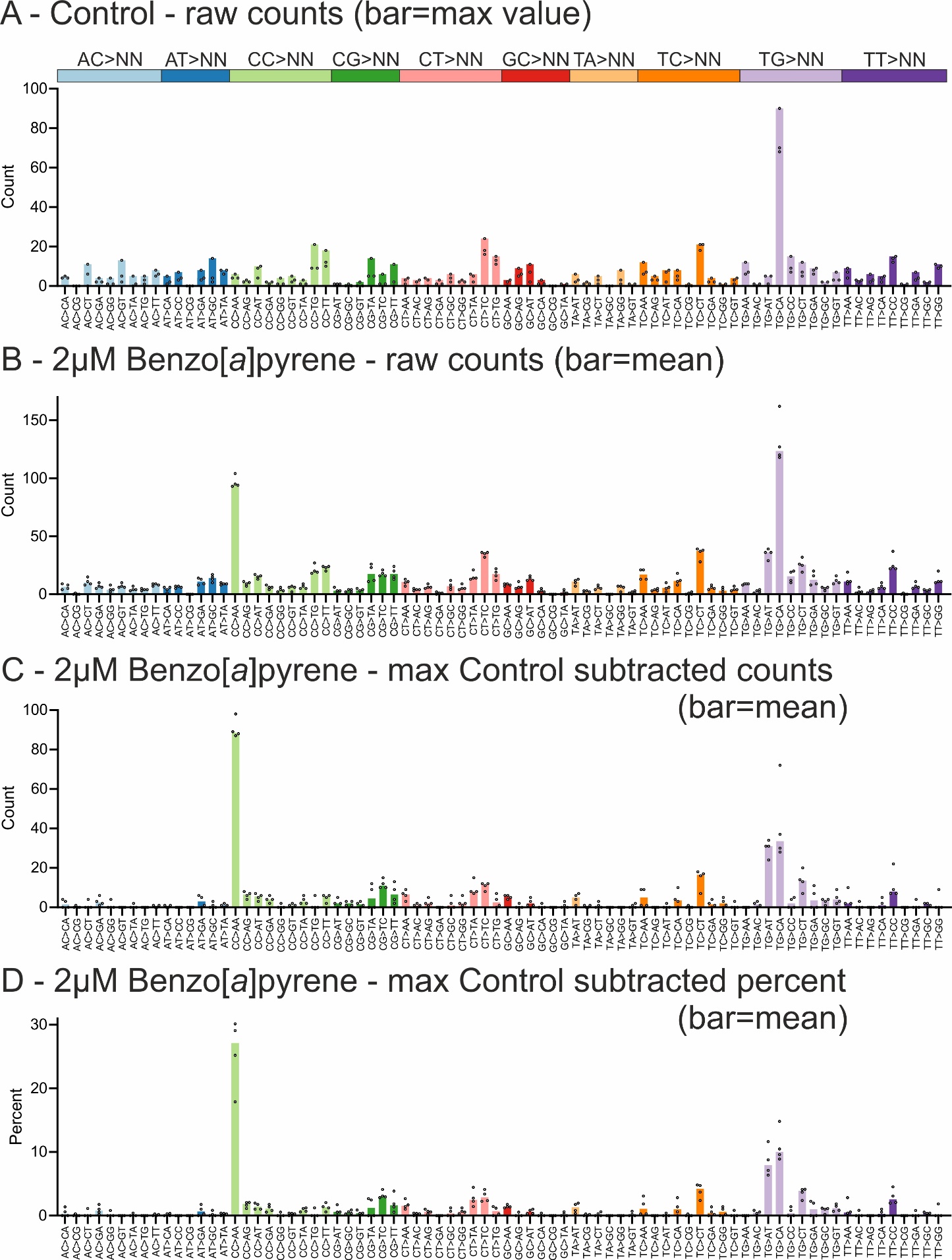
*

**Supplementary Fig. 3 – Counts and normalisation data for the DBS-BaP-NHU signature. Dots indicate the experimental replicates. The raw counts of the different mutation types are shown for (A) control and (B) BaP-exposed clones. (C) The counts from Supplementary Figure 3B with the maximum count from Supplementary Figure 3A subtracted. (D) Supplementary Figure 3C converted to percentage for comparison with other studies including COSMIC_DBS# [18]. BaP = benzo[a]pyrene; COSMIC = Catalogue of Somatic Mutations in Cancer; DBS = doublet-base substitution; NHU = normal human urothelial.**

*
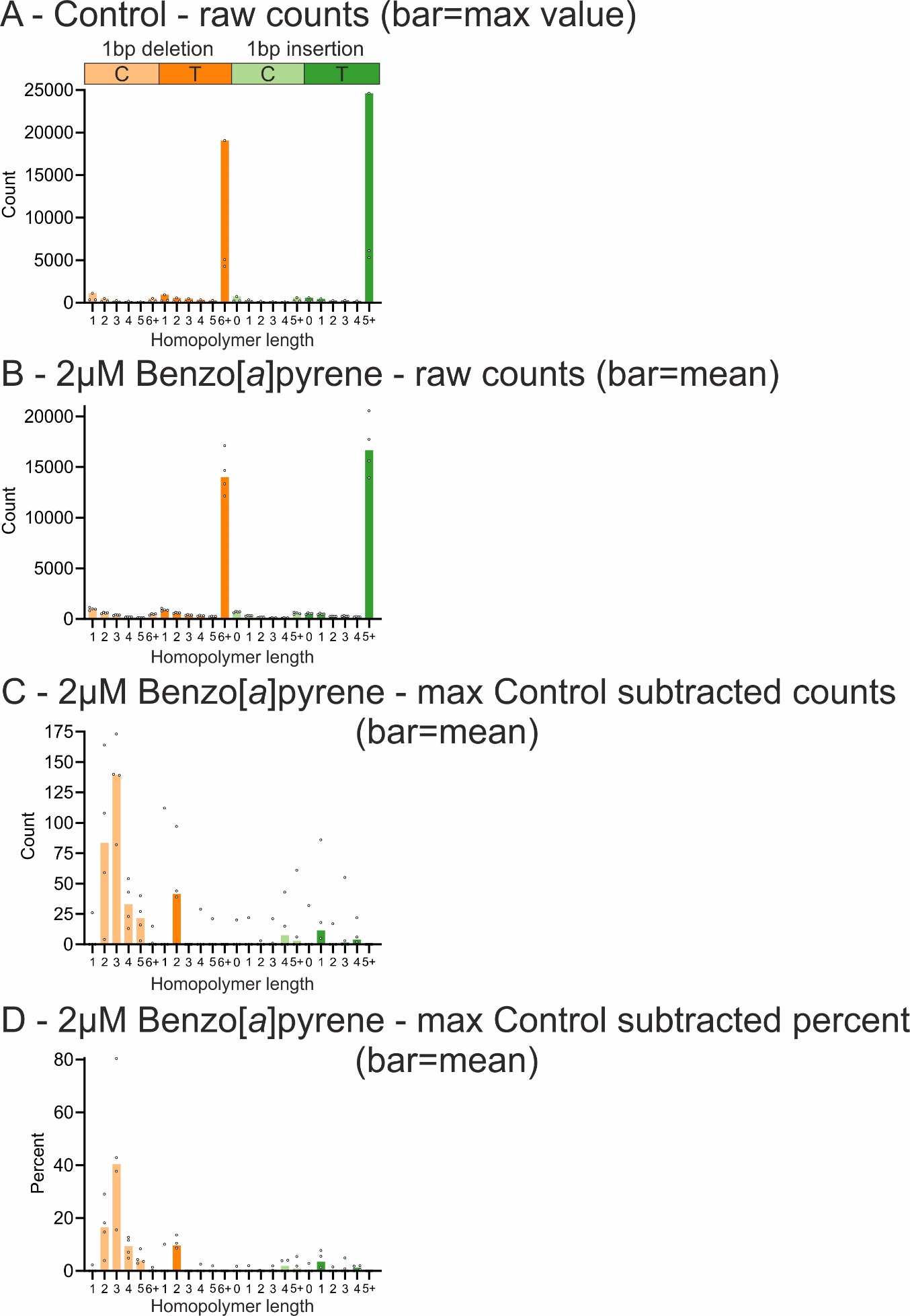
*

**Supplementary Fig. 4 – Counts and normalisation data for the ID-BaP-NHU signature. Dots indicate the experimental replicates. The raw counts of the different mutation types are shown for (A) control and (B) BaP-exposed clones. (C) The counts from Supplementary Figure 4B with the maximum count from Supplementary Figure 4A subtracted. (D) Supplementary Figure 4C converted to percentage for comparison with other studies including COSMIC_ID# [18]. BaP = benzo[a]pyrene; COSMIC = Catalogue of Somatic Mutations in Cancer; ID = insertion/deletion; NHU = normal human urothelial.**


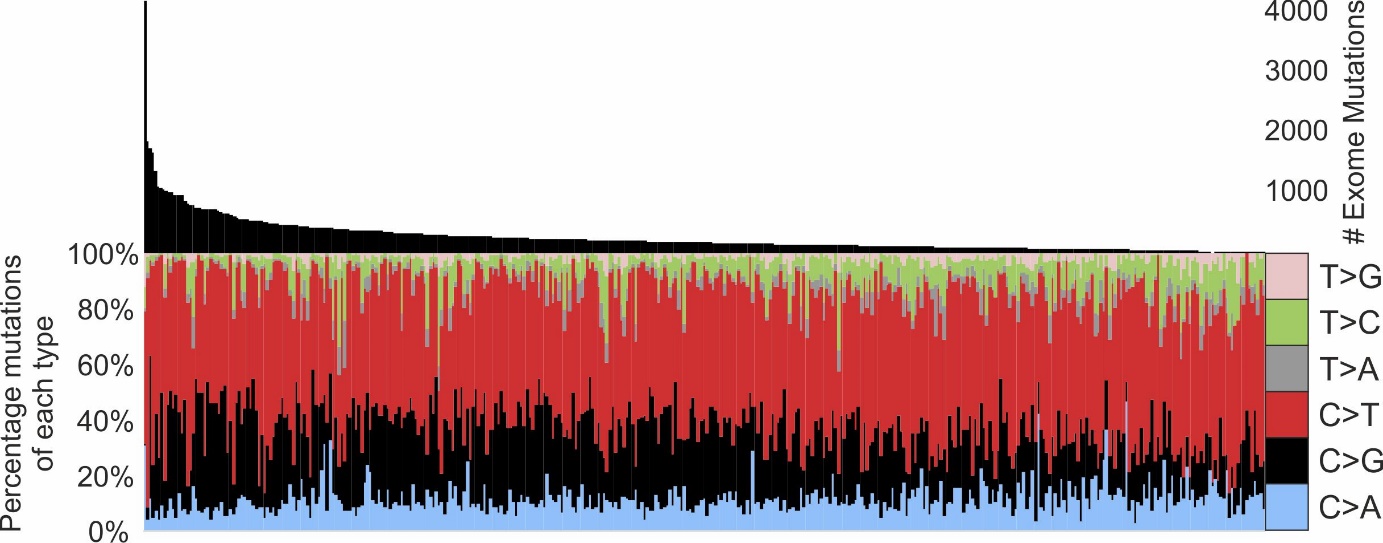


**Supplementary Fig. 5 – Frequency of specific base changes across TCGA BLCA exome sequencing data in tumours with >50 mutations in total (*n* = 393). BLCA = bladder cancer; TCGA = The Cancer Genome Atlas.**

*
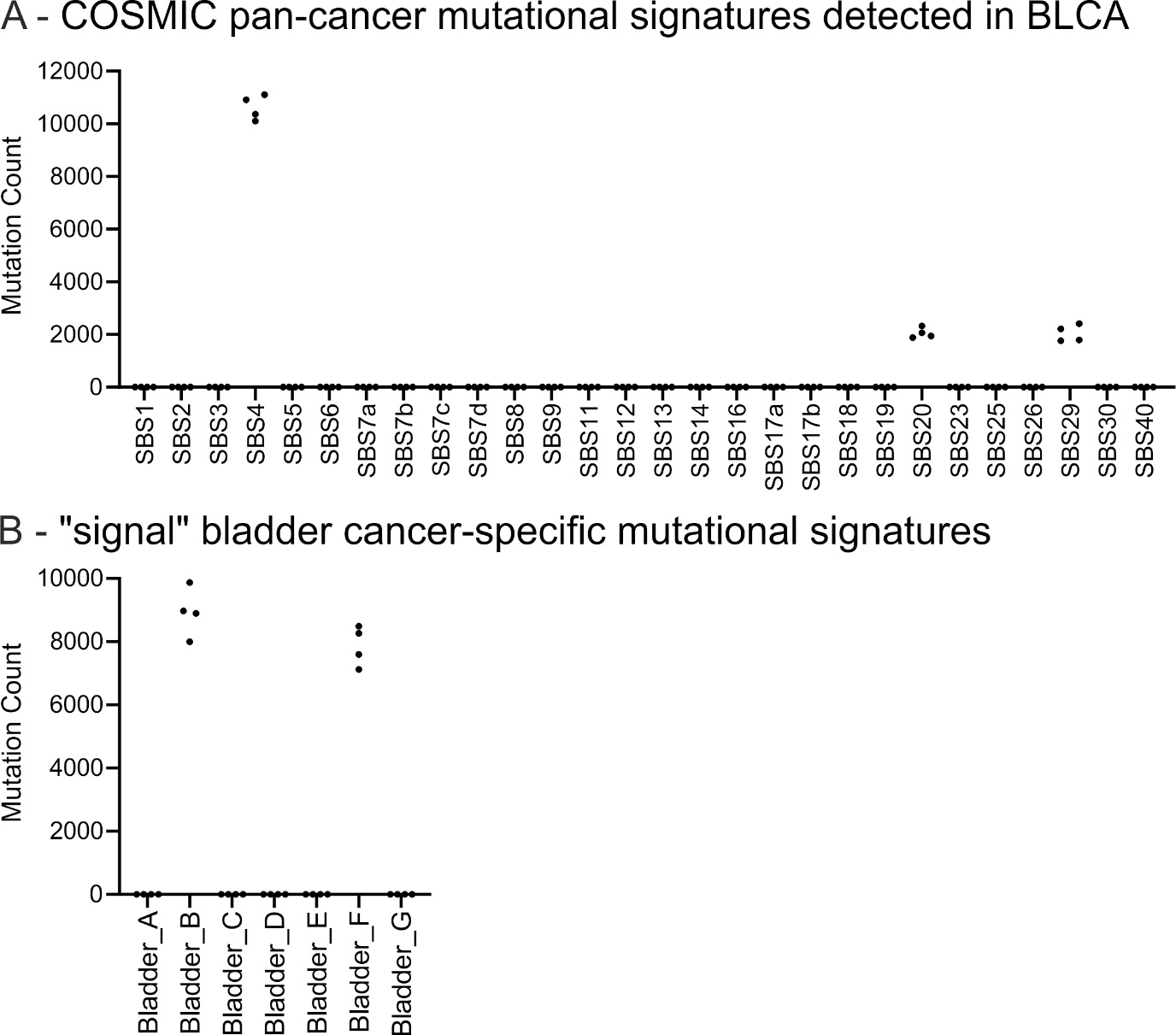
*

**Supplementary Fig. 6 – The SBS-BaP-NHU from the four independent clones was analysed using the “signal” workflow for mutational signature analysis (https://signal.mutationalsignatures.com/ [16]). (A) Comparison with the pan-cancer COSMIC v3 SBS signatures previously detected in wgDNAseq bladder cancer cohorts [18,27] found that SBS4 was the best match, likely contributing the most mutations. SBS20 and SBS29 were also potential minor contributors. SBS20 has been associated with defective DNA mismatch repair in POLD1 mutants and SBS29 with tobacco chewing [18]. No evidence of SBS2 or SBS13 contribution was noted. (B) Comparison with bladder-specific tumour signatures found that “Bladder_B” and “Bladder_F” were the closest match, with both signatures recently described as closely related to the smoking signatures of the lung, head/neck, liver, and kidney [16]. BaP = benzo[a]pyrene; BLCA = bladder cancer; COSMIC = Catalogue of Somatic Mutations in Cancer; NHU = normal human urothelial; SBS = single-base substitution; wgDNAseq = whole genome DNA sequencing.**


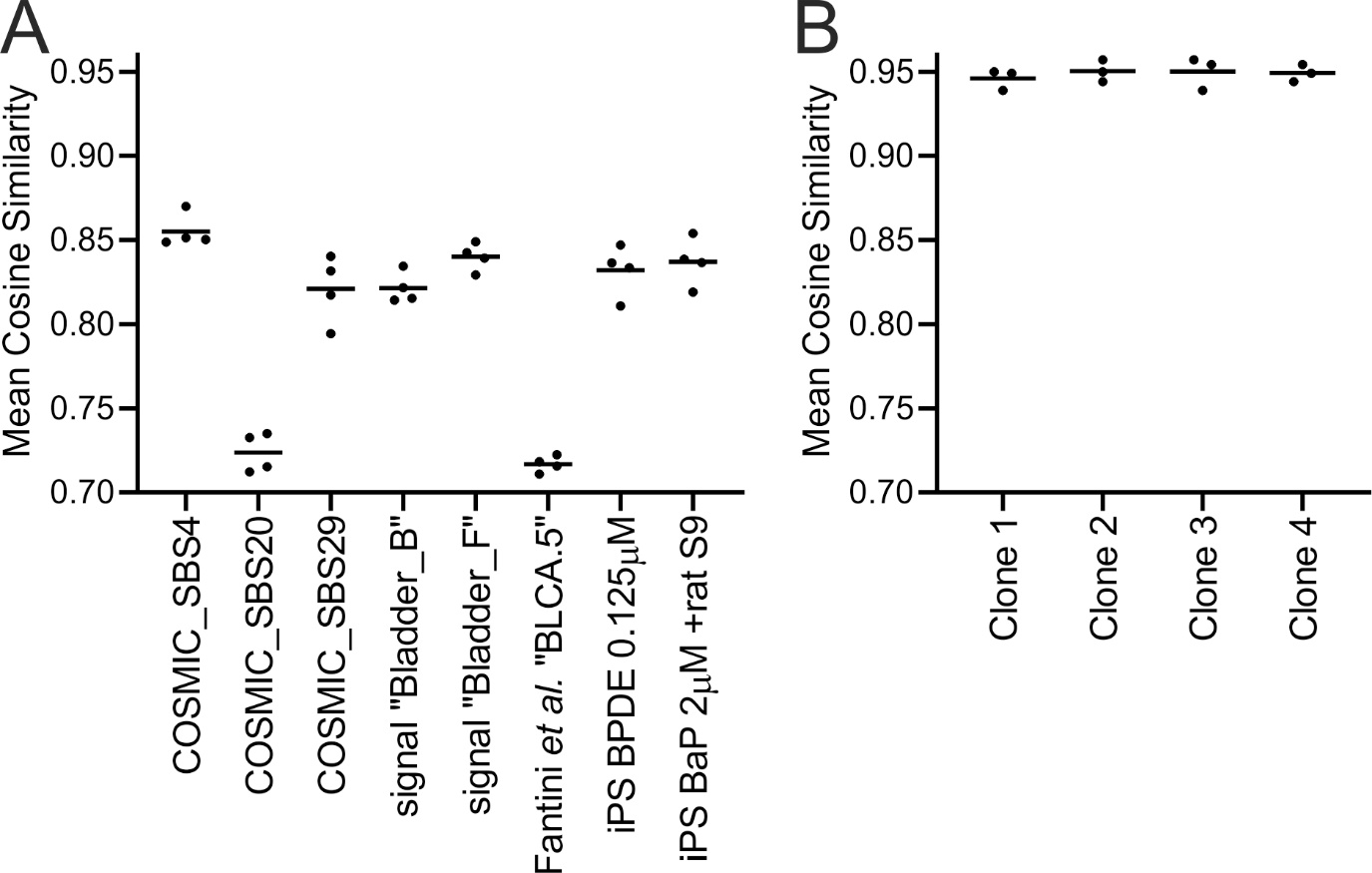


**Supplementary Fig. 7 – (A) Summary of cosine similarity tests for the SBS-BaP-NHU signatures from four independent clones against the following: (1) The pan-cancer COSMIC v3 [18] reference signatures SBS4, SBS20, and SBS29 which were likely contributors to SBS-BaP-NHU (Supplementary Fig. 6). COSMIC_SBS4 was observed in the largest (*n* = 85) wgDNAseq cohort of BLCA patients studied to date [27], although not in the smaller cohort (*n* = 23) of the COSMIC study itself [18]. COSMIC SBS20 and SBS29 have been detected more rarely in BLCA [18,27]. (2) The bladder cancer–derived signatures described in the signal workflow for mutational signature analysis (https://signal.mutationalsignatures.com/ [16]). “Bladder_B” and “Bladder_F” were the closest match, with both signatures recently described as closely related to the smoking signatures of the lung, head/neck, liver, and kidney [16]. (3) A C>A enriched signature was discovered de novo in bladder cancer by Fantini et al [19] as “BLCA.5”. Similarity between all the signatures in the C > A transversion region was very high; however, differences occur elsewhere in the signature, and especially in “BLCA.5”, which led to its lower similarity score. (4) Signatures derived from iPS cells exposed to either BaP (plus rat S9 fraction to provide metabolic activation of the procarcinogen) or BPDE (the active adduct-forming metabolite of BaP) [20]. (B) Summary of cosine similarity tests for the four replicate SBS-BaP-NHU signatures against each other shows a high degree of stability in the signature between clones. Minimum cosine similarity was >0.93. BaP = benzo[a]pyrene; BLCA = bladder cancer; BPDE = benzo(a)pyrene diol epoxide; COSMIC = Catalogue of Somatic Mutations in Cancer; iPS = induced pluripotent stem; NHU = normal human urothelial; SBS = single-base substitution; wgDNAseq = whole genome DNA sequencing.**

*
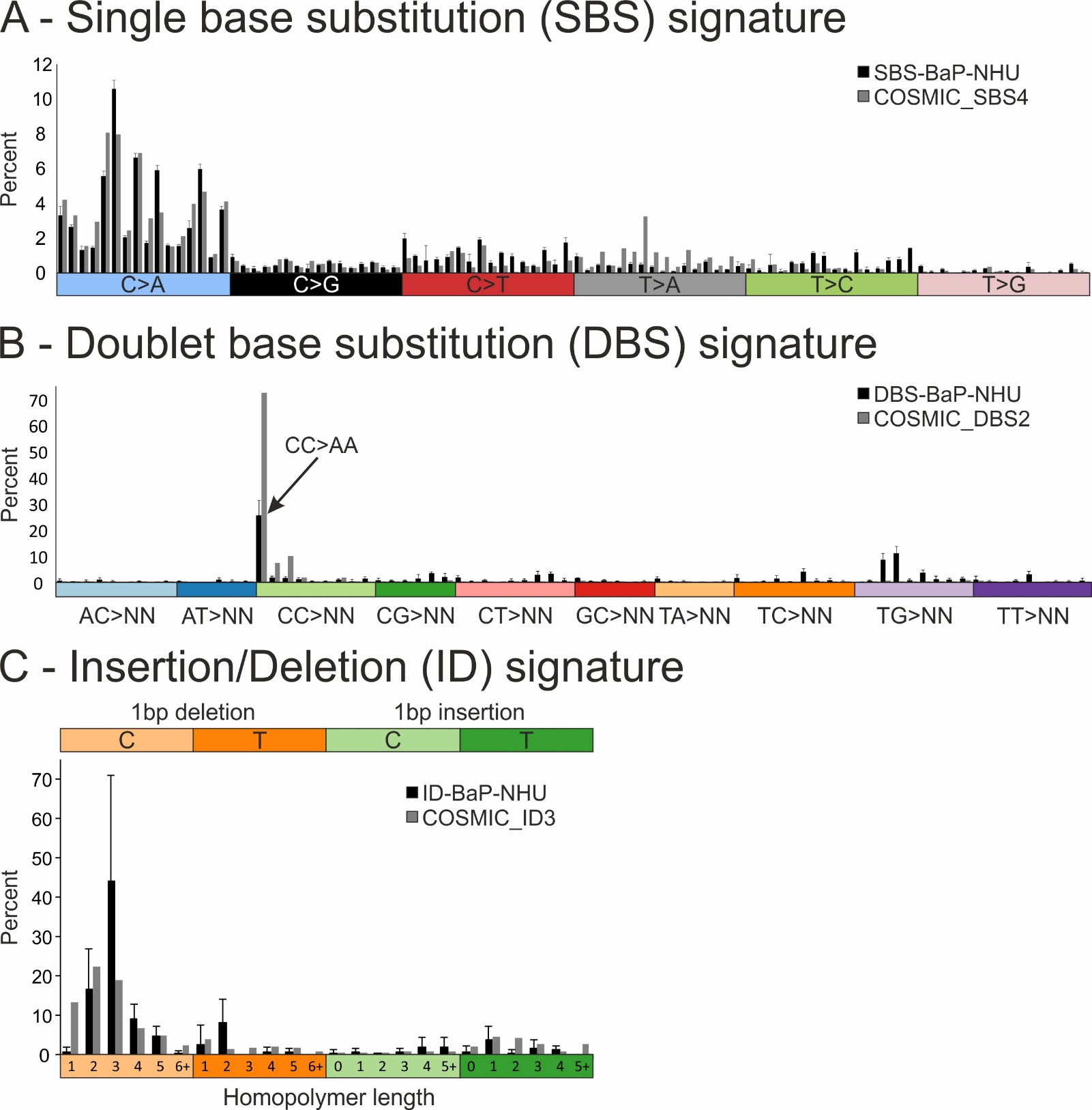
*

**Supplementary Fig. 8 – Mutational signatures observed in NHU cells exposed to BaP were highly similar to the three pan-cancer COSMIC reference smoking signatures SBS4, DBS2, and ID3 [18]. (A) Comparison of the SBS-BaP-NHU signature with COSMIC_SBS4, reflecting their mean cosine similarity score of 0.855 shown in Supplementary Fig. 7. COSMIC_SBS4 was found in the genomes of bladder cancers by a study of 85 patients [27]. (B) Similarity of the DBS-BaP-NHU signature with COSMIC_DBS2, which Alexandrov et al [18] detected in 10/10 BLCA tested. (C) Similarity of ID-BaP-NHU with COSMIC_ID3, which Alexandrov et al [18] detected in 14/24 BLCA tested. BaP = benzo[a]pyrene; BLCA = bladder cancer; COSMIC = Catalogue of Somatic Mutations in Cancer; DBS = doublet-base substitution; ID = insertion/deletion; NHU = normal human urothelial; SBS = single-base substitution.**


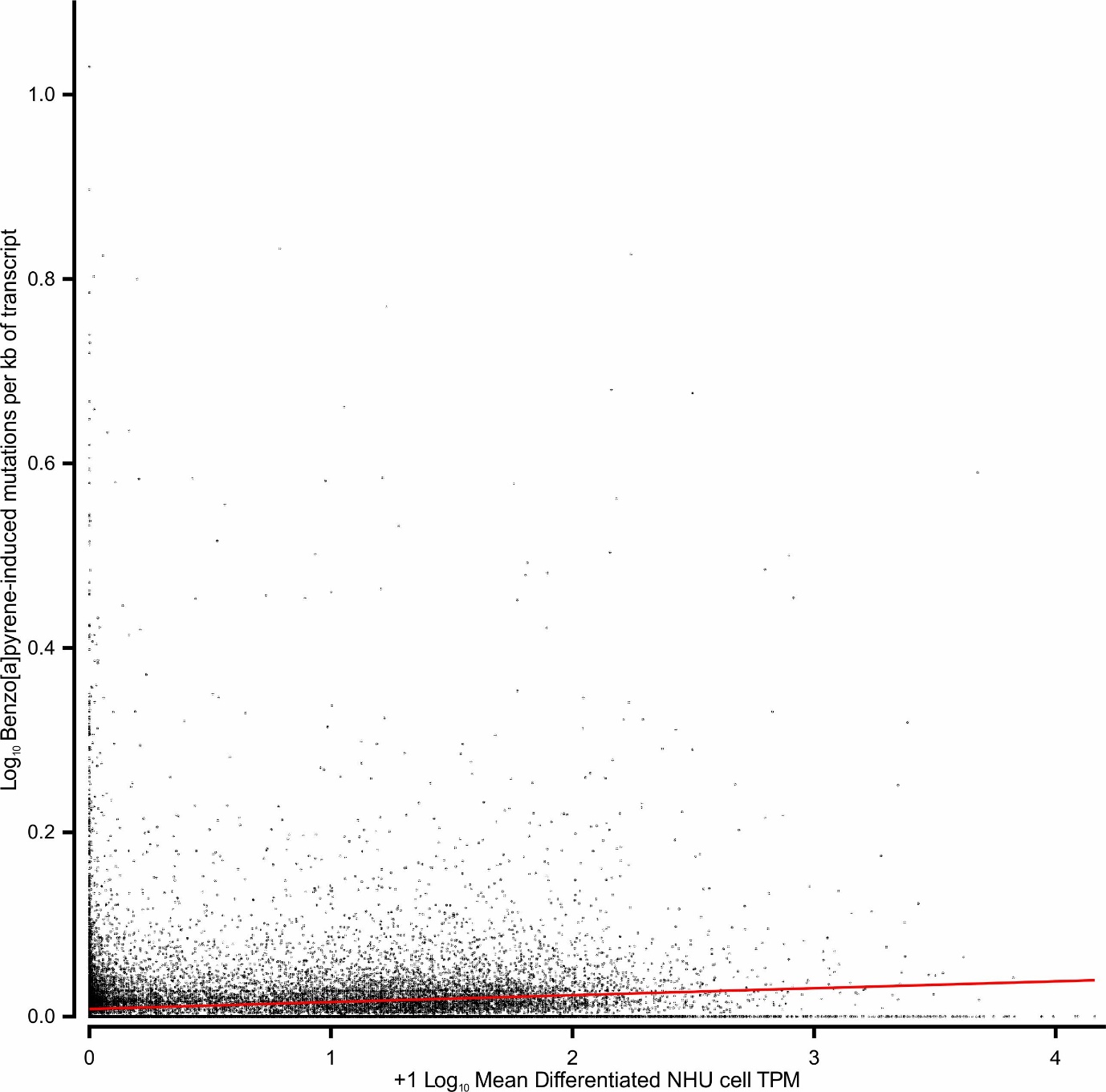


**Supplementary Fig. 9 – Scatter plot of mutation rate (summed across all BaP-exposed clones; *n* = 4) per kb of transcribed gene against gene expression in in vitro differentiated NHU cells (expressed as mean TPM). A significant positive relationship (*r* = 0.43; *p* < 0.0001) between transcription and mutation was observed by linear regression. BaP = benzo[a]pyrene; NHU = normal human urothelial; TPM = transcripts per million.**


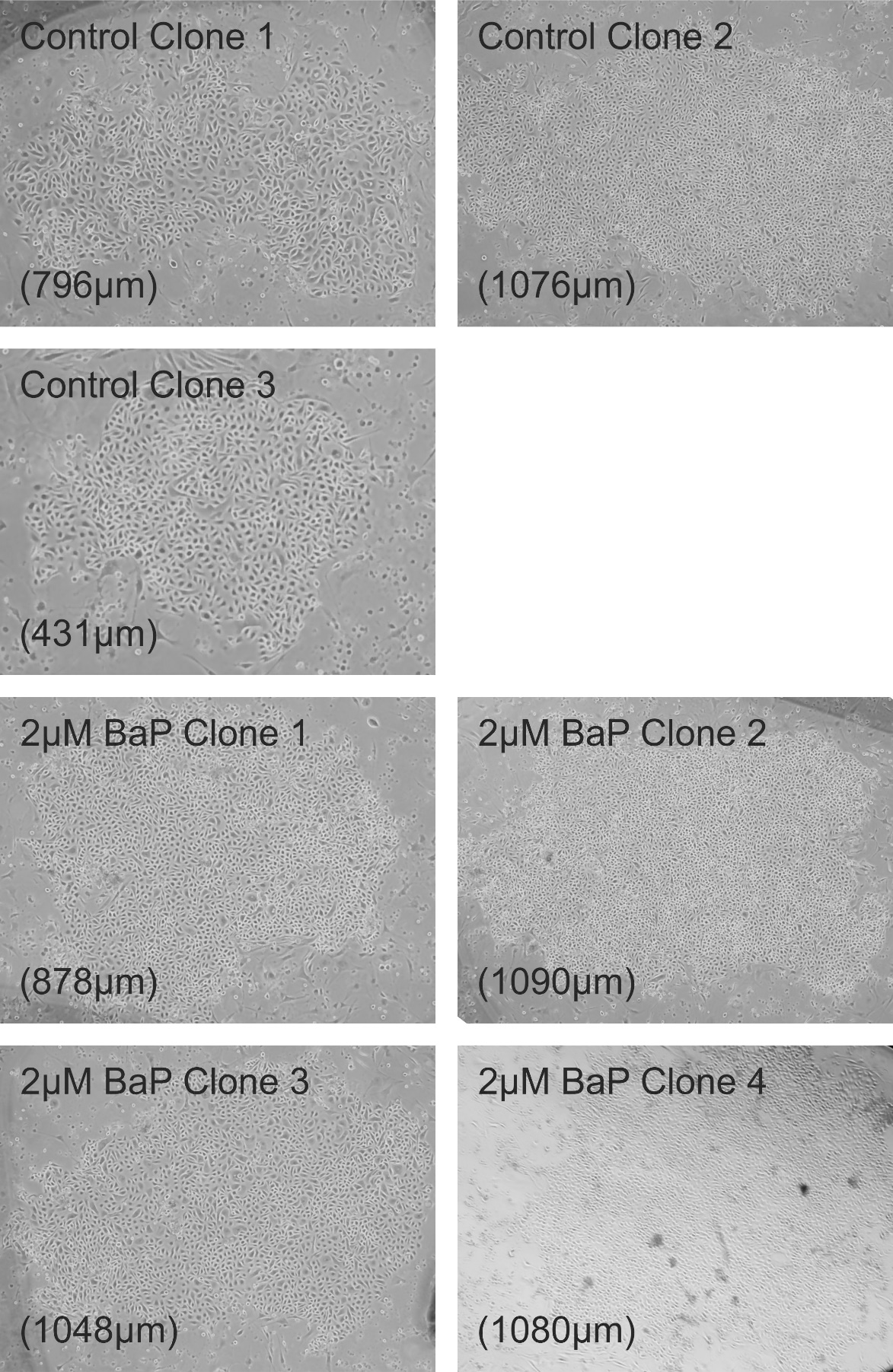


**Supplementary Fig. 10 – Phase contrast images of colonies later sent for wgDNAseq. The width of each image is included in brackets to show scale. BaP = benzo[a]pyrene; wgDNAseq = whole genome DNA sequencing.**

**Supplementary Table 1 – Genes frequently mutated in TCGA cohort of MIBC were also mutated in BaP exposed NHU cells**

| Gene | TCGA % cases mutated | Observed mutations/kb | Observed mutation no. | Expected mutation no. (if random) | *p* value |
| --- | --- | --- | --- | --- | --- |
| *TP53* | 48 | 0 | 0 | 1.61 (–0.88 to 4.10) | 0.419 |
| *KMT2D* | 28 | 0.52 | 22 | 3.48 (–0.17 to 7.13) | 2.71E-11 |
| *KDM6A* | 26 | 0.06 | 15 | 20.13 (11.33–28.93) | 0.314 |
| *ARID1A* | 25 | 0.08 | 7 | 7.20 (1.89–12.51) | 1.000 |
| *PIK3CA* | 22 | 0.02 | 2 | 7.69 (2.31–13.07) | 0.043 |
| *KMT2C* | 18 | 0.14 | 42 | 25.38 (15.68–35.08) | 2.00E-03 |
| *RB1* | 17 | 0.01 | 2 | 14.91 (7.31–22.51) | 7.83E-05 |
| *EP300* | 15 | 0.07 | 6 | 7.38 (2.00–12.75) | 0.852 |
| *ATM* | 14 | 0.01 | 1 | 12.32 (5.39–19.24) | 1.45E-04 |
| *FGFR3* | 14 | 0.06 | 1 | 1.31 (–0.92 to 3.53) | 1.000 |
| *STAG2* | 14 | 0.12 | 17 | 11.91 (5.15–18.67) | 0.145 |
| *CREBBP* | 12 | 0.04 | 7 | 13.06 (5.98–20.14) | 0.097 |
| *ELF3* | 12 | 0.15 | 1 | 0.54 (–0.88 to 1.96) | 0.419 |
| *ERBB2* | 12 | 0.02 | 1 | 3.42 (–0.20 to 7.04) | 0.277 |
| *FAT1* | 12 | 0.04 | 6 | 11.71 (5.03–18.39) | 0.106 |
| *SPTAN1* | 12 | 0.05 | 4 | 6.72 (1.66–11.79) | 0.435 |
| *KMT2A* | 11 | 0.01 | 1 | 7.59 (2.20–12.97) | 9.50E-03 |
| *ERBB3* | 10 | 0.04 | 1 | 1.98 (–0.80 to 4.77) | 1.000 |
| *ASXL2* | 9 | 0.01 | 2 | 12.25 (5.26–19.24) | 8.39E-04 |
| *CDKN1A* | 9 | 0.64 | 7 | 0.91 (–0.98 to 2.80) | 4.64E-05 |
| *ERCC2* | 9 | 0.1 | 2 | 1.74 (–0.81 to 4.28) | 0.694 |
| *FBXW7* | 8 | 0.01 | 3 | 18.07 (9.81–26.32) | 2.97E-05 |
| *TSC1* | 8 | 0.06 | 3 | 4.55 (0.30–8.80) | 0.640 |

BaP = benzo[a]pyrene; MIBC = muscle-invasive bladder cancer; NHU = normal human urothelial; TCGA = The Cancer Genome Atlas.

The table notes the number of mutations observed across all the BaP-exposed clones (*n* = 4) expressed as mutations per kilobase of gene length and absolute number. Whilst most genes accumulated mutations at a rate consistent with a random process, *KMT2D*, *KMT2C*, and *CDKN1A* (shown in red) were significantly more mutated than expected and *PIK3CA*, *RB1*, *ATM*, *KMT2A*, *ASXL2,* and *FBXW7* (shown in blue) were significantly protected from mutation.
